# A rapid phenotyping method for adult plant resistance to leaf rust in wheat

**DOI:** 10.1186/s13007-016-0117-7

**Published:** 2016-03-02

**Authors:** Adnan Riaz, Sambasivam Periyannan, Elizabeth Aitken, Lee Hickey

**Affiliations:** Queensland Alliance for Agriculture and Food Innovation, The University of Queensland, St Lucia, QLD 4072 Australia; Commonwealth Scientific and Industrial Research Organization (CSIRO) Agriculture, General Post Office Box 1600, Canberra, ACT 2601 Australia; School of Agriculture and Food Science, The University of Queensland, St Lucia, QLD 4072 Australia

**Keywords:** Wheat, Leaf rust, *Puccinia triticina*, Adult plant resistance, Accelerated growth conditions, Disease screening, Wheat breeding

## Abstract

**Background:**

Leaf rust (LR), caused by *Puccinia triticina* and is an important disease of wheat (*Triticum aestivum* L.). The most sustainable method for controlling rust diseases is deployment of cultivars incorporating adult plant resistance (APR). However, phenotyping breeding populations or germplasm collections for resistance in the field is dependent on weather conditions and limited to once a year. In this study, we explored the ability to phenotype APR to LR under accelerated growth conditions (AGC; i.e. constant light and controlled temperature) using a method that integrates assessment at both seedling and adult growth stages. A panel of 21 spring wheat genotypes, including disease standards carrying known APR genes (i.e. *Lr34* and *Lr46*) were characterised under AGC and in the field.

**Results:**

Disease response displayed by adult wheat plants grown under AGC (i.e. flag-2 leaf) was highly correlated with field-based measures (R^2^ = 0.77). The integrated method is more efficient—requiring less time, space, and labour compared to traditional approaches that perform seedling and adult plant assays separately. Further, this method enables up to seven consecutive adult plant LR assays compared to one in the field.

**Conclusion:**

The integrated seedling and adult plant phenotyping method reported in this study provides a great tool for identifying APR to LR. Assessing plants at early growth stages can enable selection for desirable gene combinations and crossing of the selected plants in the same plant generation. The method has the potential to be scaled-up for screening large numbers of fixed lines and segregating populations. This strategy would reduce the time required for moving APR genes into adapted germplasm or combining traits in top crosses in breeding programs. This method could accelerate selection for resistance factors effective across diverse climates by conducting successive cycles of screening performed at different temperature regimes.

**Electronic supplementary material:**

The online version of this article (doi:10.1186/s13007-016-0117-7) contains supplementary material, which is available to authorized users.

## Background

Wheat provides more than 20 % of the calorific intake for almost two-thirds of the human population [1]. With an expected global population of 9–10 billion by the year 2050, world food security is paramount. *Puccinia triticina* f. sp. *tritici*, which causes leaf rust (LR), is regarded one of the most geographically widespread disease of wheat and can incur yield losses ranging 10–70 % [2, 3]. It results in reduction of kernels per head, lower kernel weight, degradation in grain quality and increased costs associated with chemical control [4, 5]. In Australia, wheat diseases, including rusts, cause an estimated average annual loss of almost $913 million to the wheat industry [6]. Among the various control methods, the most profitable and sustainable disease minimization strategy is the deployment of genetically resistant cultivars [7].

To date, research around the world has resulted in designation of 73 genes for resistance to LR (i.e. *Lr*), which have been characterised and mapped to chromosomal locations [8]. Genetic resistance is broadly classed into two forms: seedling and adult-plant resistance (APR). Seedling resistance, or ‘all stage resistance’ (ASR), is typically expressed at all growth stages, conferred by a single ‘major effect’ gene often associated with a hypersensitive response and is often race specific. On the other hand, APR is typically best expressed in adult plants and often polygenic in nature, controlled by multiple ‘minor effect’ genes that may influence factors such as pustule size, infection frequency and latent period, thus commonly referred to as ‘slow rusting’ genes [9, 10]. While APR is often non-race specific, there are exceptions where some genes provide race-specific resistance (e.g. *Lr13* and *Lr37* [10, 11]) and confer a hypersensitive response (e.g. *Lr48* and *Lr49* [12]). Notably, some APR genes have been deployed for almost 100 years, such as *Sr2* and *Lr34*, which continue to provide resistance to stem rust (SR) and LR, respectively. Three well-characterized APR genes are now available to wheat breeders that appear to convey non-race specific resistance to LR (i.e. *Lr34*, *Lr46* and *Lr67*), for which useful DNA markers are also available [13, 14]. However, additional sources of resistance are needed for stacking or pyramiding in new cultivars, which will serve to protect these highly valuable genes against the rapidly evolving nature of *P. triticina*.

APR to LR is typically identified by phenotyping wheat plants at the seedling stage in the glasshouse, then subsequently evaluating adult plants in the field [10]. However, the accuracy of phenotyping in the field can be compromised by environmental factors that influence the expression of APR, such as weather patterns, inoculum pressure, sequential infection, differences in plant maturity and the presence of other diseases [15]. Further, expression of LR resistance in wheat is sensitive to temperature [16], resulting in variability across environments or years of testing [17]. Some studies have successfully evaluated APR to foliar pathogens in cereals grown under glasshouse or controlled environmental conditions (CEC) [15, 18, 19]. A key advantage is that environmental factors, such as temperature and light, can be controlled. Artificial lighting can also be used to impose an extended photoperiod or constant light to accelerate the growth of wheat plants. A plant management system providing accelerated growth conditions (AGC) could be used to speed up disease screening and plant selection.

In this study, we investigated the ability to rapidly phenotype APR to LR in wheat grown under AGC (i.e. constant light and controlled temperature). Using a panel of 21 spring wheat genotypes we compared LR response displayed by adult plants grown under AGC to levels displayed by adult plants grown in the field. We discuss opportunities to exploit this rapid phenotyping method to accelerate research and wheat breeding efforts to develop rust resistant wheat cultivars.

## Methods

### Plant materials

A panel comprising 21 spring wheat genotypes (Table 1) was used to generate a protocol for phenotyping resistance to LR in wheat grown under AGC. The panel comprised a selection of standards, cultivars and breeding lines from Australia, the International Center for Agriculture Research in the Dry Areas (ICARDA), and the International Maize and Wheat Improvement Center (CIMMYT).

**Table 1:** Name, pedigree, breeding program and leaf rust resistance genes present in 21 spring wheat genotypes

Genotypes	Pedigree	Type	Resistance genes		Breeding program	Source^a^
			Seedling	APR		
Thatcher	MARQUIS/IUMILLO DURUM//MARQUIS/KANRED	Cultivar	–^b^	–	North America	[35]
Avocet	THATCHER-*AGROPYRON ELONGATUM* TRANSLOCATION/3* PINNACLE//WW15/3/EGRET	Cultivar	–	*Lr13*	Australia	[36]
Avocet + *Lr34*	AVOCET NEAR ISOGENIC LINE *LR34*	Near isogenic line	–	*Lr34*	Near Isogenic Line	[37]
Avocet + *Lr46*	AVOCET NEAR ISOGENIC LINE *LR46*	Near isogenic line	–	*Lr46*	Near Isogenic Line	[37]
Dharwar Dry	DWR39/C306//HD2189	Cultivar	–	–	India	–
Drysdale	HARTOG*3/QUARRION	Cultivar	*Lr1*	*Lr13*	Australia	[27]
Janz	3AG3/4*CONDOR//COOK	Cultivar	*Lr24*	*Lr34*	Australia	[27]
Lang	QT3765/SUNCO	Cultivar	*Lr24*	*Lr34*	Australia	[27]
EGA Gregory	PELSART/2*BATAVIA	Cultivar	*Lr1, Lr3a, Lr23*	*Lr13, Lr34*	Australia	[27]
EGA Wylie	QT2327/COOK//QT2804	Cultivar	*Lr17a*	*Lr34*	Australia	[27]
FAC10-16-1	10CB-F/W234	Breeding line	–	–	ICARDA	–
Mace	WYALKATCHEM/STYLET//WYALKATCHEM	Cultivar	*Lr23*	*Lr13, Lr37*	Australia	[27]
RIL114	UQ01484/RSY10//H45	Breeding line	–	–	Australia	–
SB062	SERI M82/BABAX	Breeding line	–	–	Australia	–
Scout	SUNSTATE/QH71-6//YITPI	Cultivar	*Lr1*	*Lr37*	Australia	[27]
Suntop	SUNCO/2*PASTOR//SUN436E	Cultivar	–	–	Australia	–
SeriM82	KAVKAZ/(SIB)BUHO//KALYANSONA/BLUEBIRD	Breeding line	*Lr23, Lr26*	–	CIMMYT	–
Zebu	–	Cultivar	*Lr26*	–	CIMMYT	[27]
ZWB10-37	TACUPETOF2001/BRAMBLING//KIRITATI	Breeding line	–	–	CIMMYT	–
ZWW10-128	ESDA/KKTS	Breeding line	–	–	CIMMYT	–
ZWW10-50	ONIX/4/MILAN/KAUZ//PRINIA/3/BAV92	Breeding line	–	–	CIMMYT	–

^a^Study reporting the status of leaf rust resistance genes

^b^A dash (–) indicates data is unavailable or unknown

### Rust screening: seedling stage

The panel was evaluated for resistance to LR at the seedling stage in a glasshouse at The University of Queensland, St Lucia, Queensland, Australia. Seeds were imbibed for 24 h at room temperature and were placed in a refrigerator (4 °C) for 48 h to encourage synchronous germination across genotypes. Germinated seeds were transplanted into 140 mm ANOVApot^®^ pots filled with a potting media consisting of composted pine bark fines (0–5 mm) (70 %) and coco peat (30 %) with a pH ranging 5.5–6.5. Slow release Osmocote^®^ fertilizer was applied at a rate of 2 g per pot. Each pot contained four different positions (i.e. positions 1–4 clockwise from the pot tag), where each position contained four germinated seeds of the same genotype clumped together. Each genotype was replicated three times in a completely randomized design. Plants were grown at a temperature regime of 22/17 °C (day/night) and a natural 12 h diurnal photoperiod. After 10 days, (i.e. two-leaf stage) plants were inoculated with *P. triticina* pathotype (*pt*) 104-1,2,3,(6),(7),11,13. This pathotype evolved from pathotype 104-1,2,3,(6),(7),11 via a single step mutation on wheat carrying the resistance gene *Lr24* and was first reported in Australia in 2000 [20]. It currently occurs in wheat production regions throughout the east coast of Australia. The rust isolate used in this study was developed using a single spore culture technique and spores increased using susceptible wheat cultivar Morocco. The inoculum was prepared by suspending urediniospores in light mineral oil (Isopar 6) at a rate of 0.005 g/ml. Inoculum at the concentration of 6 × 10^5^ spores/ml was applied to the leaves of wheat plants using an air brush (IWATA power jet lite^®^). Plants were then lightly misted with deionized water and placed in a dew chamber maintained at 100 % humidity using an ultrasonic fogger. After 18 h of incubation, plants were removed from the dew chamber and returned to the glasshouse for subsequent disease development. Twelve days post-inoculation seedlings were assessed for infection type (IT) using the 0–4 Stakman scale [21]. Genotypes that displayed an IT of <3 were considered resistant.

### Rust screening: adult plant stage

In total, three adult plant experiments were conducted using the panel. Two phenotyping experiments, namely, “adult plant integrated” and “adult plant independent” were conducted under AGC, while phenotyping in the field was conducted in a disease screening nursery.

#### Adult plant experiment 1: integrated method under AGC

Following assessment of disease response at the seedling stage (as describe above), the plants were transferred to a fully-enclosed temperature controlled growth facility (dimensions 5 m × 6 m). The growth facility is fitted with 20 low-pressure sodium vapor lamps (400 watt each) generating 400–550 µmol M^−2^ S^−1^ photosynthetically active radiation (PAR) at pot height and 900 µmol M^−2^ S^−1^ at adult plant height (i.e. about 45 cm above pot level). AGC was achieved by adopting constant (i.e. 24 h) light [19] and a 12 h cycling temperature regime of 22/17 °C. Pots were positioned on a bench according to a completely randomized design in a stainless steel tray (240 × 90 × 10 cm). Plants were grown for 2 weeks under AGC, and then re-inoculated with a suspension of LR urediniospores (*pt* 104-1,2,3,(6),(7),11,13), as described above. Prior to inoculation, the developmental growth stage (GS) was recorded for each plant using the Zadoks decimal code scoring system [22]. Twelve days post-inoculation IT was recorded for different leaves (i.e. flag, flag-1 and flag-2) on the primary/main tiller of each plant using the 0–4 Stakman scale. Genotypes displaying an IT of <3 were considered resistant.

#### Adult plant experiment 2: independent method under AGC

As a control, a new batch of plants were sown for the panel and grown from day one under AGC. Environmental conditions and experimental design was consistent with adult plant experiment 1 (above). Three weeks after sowing, the majority of genotypes achieved the adult plant stage and were inoculated with *pt* 104-1,2,3,(6),(7),11,13, as outlined above. Prior to inoculation, the GS for all plants was recorded using the Zadoks scale. Twelve days later, plants were assessed for IT using the Stakman scale.

#### Adult plant experiment 3: in the field

The panel of wheat genotypes was evaluated for response to LR in the field at Redlands Research Facility, Queensland, Australia, from July to October 2014. Six seeds of each genotype was sown as un-replicated hill plots. The susceptible genotype Morocco was used as a disease spreader in the field nursery, where two rows of Morocco were sown between each bay compromising two rows of hill plots. LR epidemics were initiated by transplanting Morocco seedlings infected with *pt* 104-1,2,3,(6),(7),11,13 (as outlined above) into the field among the spreader rows about 5 weeks after sowing. The LR epidemic was promoted with sprinkler irrigation applied in the late evenings when temperatures were favorable for infection and high humidity and low winds at night were expected. Once the epidemic had sufficiently developed on LR standards to allow a clear differentiation between susceptible and resistant genotypes, disease response was assessed on a whole plot basis using the modified Cobb scale [23]. Multiple disease assessments were conducted from late tillering/stem elongation to early grain filling (i.e. 70, 77, 86 and 96 days after sowing; DAS). Host response and disease severity data were used to calculate coefficient of infection (CI), as per Loegring et al. [24]. Genotypes that displayed a LR response from resistant (R) to moderately resistant-moderately susceptible (MRMS) were considered resistant.

### Statistical analysis

For experiments performed under controlled conditions, LR response was evaluated using the 0–4 Stakman scale, which encompasses both numbers (e.g. 0, 1…4) and symbols (e.g. ;, +). This data was converted to the 0–9 scale, where 0 = immune and 9 = very susceptible, using a conversion table [25]. The IT were converted as follows: 0;, ;n, ;, 1−, 1, 1+, 2−, 2, 2+, 2++, 3−, 3 3+, 3++ and 4 were coded as 0, 0.5, 1, 2.5, 3, 3.5, 4, 5, 6, 6.5, 7, 8, 8.5 and 9 respectively. For heterogeneous ITs, each score was converted individually to the 0–9 scale and the average calculated. The converted datasets were then used for further statistical analysis.

Data analysis was performed using GenStat 17.1 © 2000–2015 VSN International Ltd. Analysis of variance (ANOVA) was performed using the converted data for experiments including; seedling, adult plant integrated and adult plant independent. Mean disease response and standard error means (SEM) for each genotype were calculated for comparison of disease reactions.

Regression analyses were performed to investigate the correlation between phenotypes observed for the different experiments and to determine which leaf (i.e. flag, flag-1 and flag-2) under AGC provided the best estimate for LR response in the field for each disease assessment (i.e. 70, 77, 86 and 96 DAS). For the field dataset, CI values obtained from the un-replicated hill plots were used for regression analyses. The CI values were divided by 10 to convert to the 0–9 scale. The converted scores were used in comparison of mean LR response and principle component analysis. To investigate trends in disease response displayed by genotypes across multiple experiments, a principle component analysis (PCA) was performed and results visualized in the form of a biplot. This was performed using the following phenotype datasets: (1) seedling, (2) adult plant integrated, (3) adult plant independent, and (4) adult plant in the field (i.e. fourth assessment at 96 DAS). The disease response for flag-2 was used for both adult plant experiments conducted under AGC.

## Results

### Rust screening: seedling stage

**Fig. 1:**
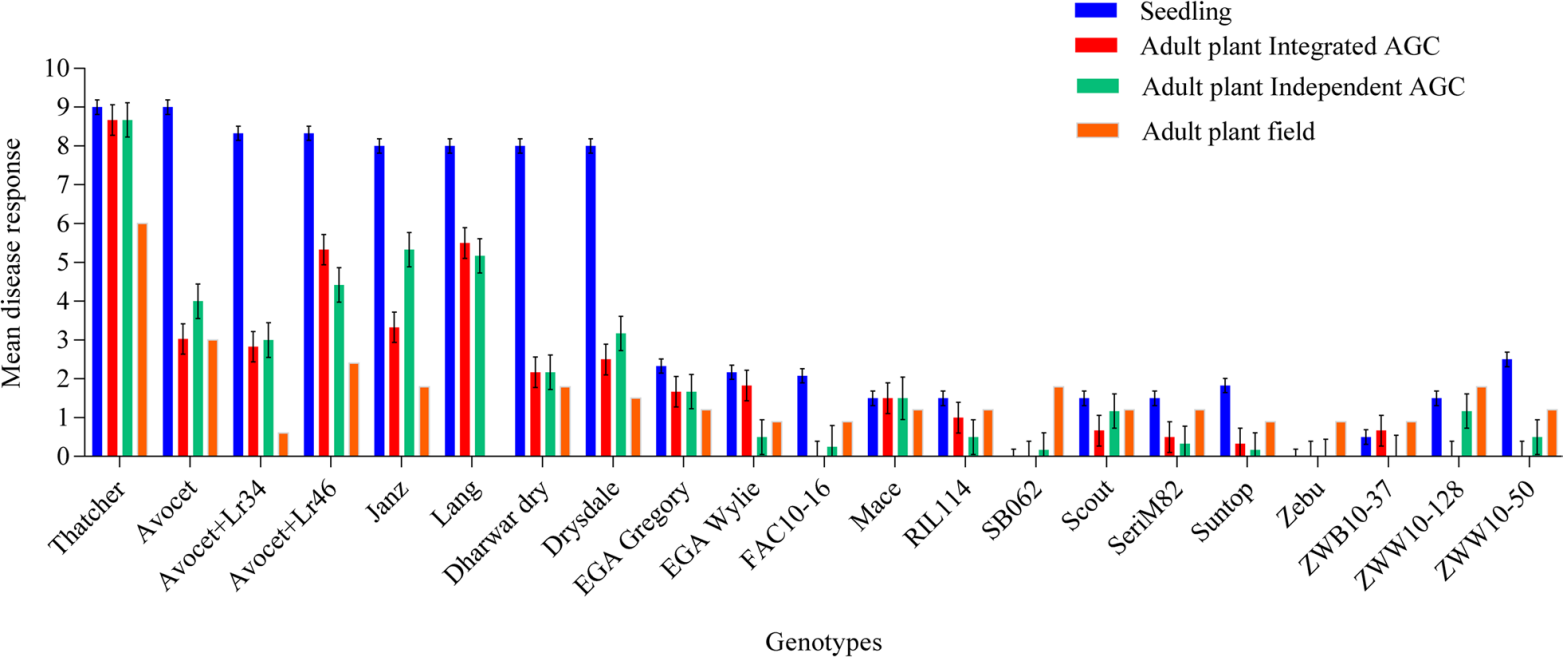
Mean leaf rust response for the panel of 21 spring wheat genotypes evaluated in the following experiments: seedling (standard glasshouse), adult plant integrated and adult plant independent under accelerated growth conditions (AGC), and in the field. The disease response for the seedling and adult plant AGC experiments was collected using the 0–4 scale and converted to the 0–9 scale (displayed). Whereas, the disease response in the field was collected using the modified Cobb scale, which was used to calculate coefficient of infection, and was converted to the 0–9 scale (displayed)

Of the 21 spring wheat genotypes in the panel, 8 displayed susceptibility, while 13 displayed resistance to LR pathotype 104-1,2,3,(6),(7),11,13 at the seedling growth stage (Fig. 1). Thatcher, Avocet, Avocet + *Lr34*, Avocet + *Lr46*, Dharwar dry, Drysdale, Lang and Janz displayed susceptibility with characteristic symptoms of large uredia without chlorosis (i.e. mean disease responses ranging 7–9; Fig. 1). The susceptible standard, Thatcher, lacks effective LR resistance genes and displayed a mean disease response of 9.0. Notably, Avocet carries a race specific APR gene *Lr13* [26] and displayed seedling susceptibility (9.0; Fig. 1). The Indian cultivar Dharwar dry, previously uncharacterized for LR resistance genes, also displayed susceptibility (8.0). Drysdale carries *Lr1* (Table 1), which is ineffective against the pathotype used in this study [27] and displayed a susceptible response (8.0; Fig. 1). Janz and Lang displayed susceptibility at the seedling stage (i.e. 8.0; Fig. 1); both genotypes carry *Lr24* and *Lr34* (Table 1). The seedling gene *Lr24* is ineffective against *pt* 104-1,2,3,(6),(7),11,13 [20], whereas *Lr34* is an APR gene and best expressed at adult plant growth stage [13]. Based on the Stakman scale, the IT of seedling susceptible genotypes range from 3 to 4 (see Additional file 1: Table S1).

EGA Gregory carries *Lr1*, *Lr3a*, *Lr13*, *Lr23* and *Lr34* (Table 1) and displayed a moderately resistant (MR) response (2.3; Fig. 1). The seedling resistance displayed by EGA Gregory was likely due to *Lr13*, as both *Lr1,Lr3a* and *Lr23* are ineffective against the pathotype. The MR response displayed by Mace (1.5; Fig. 1) was also likely due to *Lr13* and *Lr37* (Table 1)*. Lr13* and *Lr37* are APR genes and are effective against the pathotype used in this study (Table 1). Previous studies have reported early expression of *Lr13* at the seedling stage [28]. Scout carries *Lr1* and *Lr37* (Table 1), where *Lr1* is ineffective against this pathotype, while *Lr37* is effective. Scout displayed a MR response (1.5) at the seedling stage, which could be due to an uncharacterized seedling resistance or early expression of *Lr37* at the seedling stage (Fig. 1) [29]. EGA Wyile carries *Lr17a* and *Lr34* (Table 1) and displayed a MR response (2.2; Fig. 1), as the pathotype used in this study is avirulent on *Lr17a*. SeriM82 and Zebu carry *Lr26* and both displayed a highly resistant response (1.5 and 0.0, respectively; Table 1 and Fig. 1). The previously, uncharacterized ICARDA line (FAC10-16-1) displayed a MR response (2.1; Fig. 1). Other genotypes previously uncharacterised for LR resistance genes, including RIL114, Suntop, SB062, ZWB10-37 and ZWW10-128 depicted high levels of resistance with mean disease response ranging 0–1.5 (Fig. 1). Based on the Stakman scale, the IT of the seedling resistant genotypes ranged from 0; to 12+; (see Additional file 1: Table S1).

### Rust screening: adult stage under AGC

In both adult plant experiments performed under AGC (i.e. integrated and independent), 20 of the 21 genotypes in the panel displayed varying levels of resistance (Fig. 1). In both experiments, Thatcher displayed a very susceptible (VS; 9.0) response with urediniospores freely sporulating on leaves (Fig. 1). Avocet displayed a resistant-moderately resistant (RMR) response with a mean disease response ranging 3–4 (Fig. 1). As mentioned earlier, Avocet carries race specific APR gene *Lr13*, which is effective against the pathotype used in this study. In the Avocet background, resistance to LR was slightly enhanced with the addition of *Lr34* and *Lr46* (i.e. *Avocet* + *Lr34* and *Avocet* + *Lr46*), which are considered multi-resistance APR genes (Fig. 1). Avocet + *Lr34* displayed a RMR response with mean disease response ranging 2.8–3.0 and Avocet + *Lr46* displayed a MR response, ranging 4.4–5.3 in the adult plant independent and integrated experiments, respectively. On the Stakman scale, the IT displayed by Avocet + *Lr34* and Avocet + *Lr46* ranged; n12-(independent) to 12-(integrated), where pustules were smaller in comparison to Avocet and some necrosis in case of *Lr34* (see Additional file 1: Table S1). The Indian cultivar Dharwar dry displayed a resistant response in both AGC experiments (Fig. 1). Dharwar dry has not been previously characterized for rust resistance genes, thus the underlying genes are unknown. Drysdale carries *Lr1* along with race specific APR *Lr13* and displayed resistance (Table 1; Fig. 1). Both Janz and Lang carry *Lr24* and *Lr34* in combination (Table 1) however *Lr24* was not effective against the pathotype used in this study. These genotypes displayed a MRMS response, likely due to expression of APR gene *Lr34* (Fig. 1). The mean disease response for Janz and Lang was 3.3 and 5.5 in adult plant integrated experiment, respectively, and displayed similar responses in the adult plant independent experiment (i.e. 5.3 and 5.2, respectively; Fig. 1). EGA Gregory (1.7) and Mace (1.5) displayed a resistant response in both AGC experiments (Fig. 1). EGA Gregory carries *Lr1*, *Lr3a*, *Lr13*, *Lr23* and *Lr34* and Mace carries *Lr1*, *Lr23*, and *Lr37* (Table 1). The LR *pt* 104-1,2,3,(6),(7),11,13 is virulent on both *Lr1,Lr3a* and *Lr23*, but avirulent on APR genes *Lr13*, *Lr34* and *Lr37*. Thus, resistance displayed at adult growth stages by EGA Gregory and Mace is likely a combination of these genes. Scout displayed resistance (1.5) (Fig. 1), most likely attributable to *Lr37* (Table 1). EGA Wylie displayed a highly resistant (HR) response in the integrated (1.8) and independent (0.5) AGC experiments (Fig. 1). This was most likely a result of the combined effect of seedling gene *Lr17a* and APR gene *Lr34* (Table 1). SeriM82 depicted a HR response in AGC experiments (Fig. 1), most likely due to the presence of seedling gene *Lr26* (Table 1). Genotypes previously uncharacterised for LR resistance genes (including SB062, RIL114, Suntop, Zebu, ZWW10-50, ZWW10-37, ZWW10-128 and FAC10-16-1) displayed high levels of resistance in AGC experiments (Fig. 1), indicating effective resistance to the pathotype used in this study. The detailed IT for these genotypes is provided in Additional file 1: Table S1. Overall, comparison of datasets from the integrated and independent experiments performed under AGC revealed only minor differences in infection and response types displayed by the panel of genotypes. Genotypes either displayed the same response or it varied within only one response type across both experiments. For instance, Drysdale displayed a RMR response in the independent experiment, but displayed R response in the integrated experiment (Fig. 1; Additional file 1: Table S1). The GS of plants evaluated under AGC ranged between GS25-45 and GS23-43 (i.e. tillering to booting stage) for the integrated and independent experiments, respectively (Table 2).

**Table 2:** Zadoks growth stages for the panel of 21 spring wheat genotypes at inoculation under accelerated growth conditions

Genotypes	Growth stage at inoculation	
	Adult plant integrated	Adult plant independent
Thatcher	31	37
Avocet	33	43
Avocet + *Lr34*	34	41
Avocet + *Lr46*	39	41
Dharwar dry	37	31
Drysdale	37	25
Janz	32	31
Lang	31	31
EGA Gregory	30	25
EGA Wylie	32	25
FAC10-16-1	33	25
Mace	30	25
RIL114	45	41
SB062	32	26
Scout	37	25
SeriM82	33	37
Suntop	39	37
Zebu	28	26
ZWB10-37	30	31
ZWW10-50	37	26
ZWW10-128	37	26

### Rust screening: in the field

All genotypes in the panel displayed varying levels of resistance to LR, with the exception of Thatcher, which consistently displayed a susceptible response (60 S; Additional file 1: Table S1). Avocet displayed a MRR response for the first three disease assessments; however on the fourth assessment, Avocet displayed a 50 MRMS response (Additional file 1: Table S1). In the Avocet background, the APR gene *Lr34* (i.e. Avocet + *Lr34*) displayed a 20 MRR response, while Avocet + *Lr46* displayed a MRMS response (40 MRMS; Additional file 1: Table S1). Dharwar dry displayed a MRMS response (30 MRMS), likely due to the presence of uncharacterised APR gene(s) (see Additional file 1: Table S1). Drysdale displayed a MRR response in the field, likely due to race specific APR *Lr13* (50 MRR). Janz carries *Lr24* and *Lr34* in combination and displayed the MRMS response (30 MRMS). As the pathotype used in this study is virulent on *Lr24*, the resistance displayed by Janz is likely due to *Lr34* (see Additional file 1: Table S1). CIMMYT lines (ZWW10-128 and SB062) both displayed a MRR response in the first three disease assessments, however, on the fourth assessment, each was considered MRMS (30 MRMS). ICARDA breeding line FAC10-16-1 was considered RMR (30 RMR) in the field. Other genotypes, such as EGA Gregory, EGA Wyile, Mace, Scout, RIL114, Suntop, Zebu, ZWW10-50, and ZWW10-37, displayed high levels of resistance (i.e. MRR) in the field with mean disease response ranging 30–40 MRR (see Additional file 1: Table S1). Lang failed to germinate in the field. The detailed host response and disease severity data is provided in Additional file 1: Table S1.

### Adult plant assessment under AGC is predictive of field response

Based on regression analyses, the LR response for different leaves showed very good correspondence across the two adult plant AGC experiments: R^2^ = 0.90 (flag), 0.88 (flag-1) and 0.96 (flag-2). Despite all leaves showing good correspondence, the flag-2 leaf was considered to provide the most consistent LR response across AGC experiments. Regression analysis was also performed using data from the adult plant integrated AGC experiment and the field. The highest correlation was found for the response displayed by the flag-2 leaf versus the fourth (final) disease assessment in the field (R^2^ = 0.77; Table 3). Correlations for the other leaves (flag and flag-1) corresponding with the four disease assessments ranged between 0.43–0.57 and 0.63–0.76, respectively (Table 3).

**Table 3:** Results from regression analysis (R^2^ values) for the panel of 21 spring wheat genotypes evaluated for leaf rust response in the adult plant integrated experiment versus the field

Leaf number	Number of observations (n)	Days after sowing (DAS)			
		70	77	86	96
Flag	15	0.55	0.43	0.51	0.57
Flag-1	19	0.76	0.63	0.71	0.73
Flag-2	19	0.76	0.60	0.74	0.77

Regression analysis was performed for the disease response displayed by each leaf under accelerated growth conditions (i.e. Flag, Flag-1 and Flag-2) in comparison to the field response observed for each of the four assessment dates (i.e. 70, 77, 86 and 96 days after sowing, DAS)

**Fig. 2:**
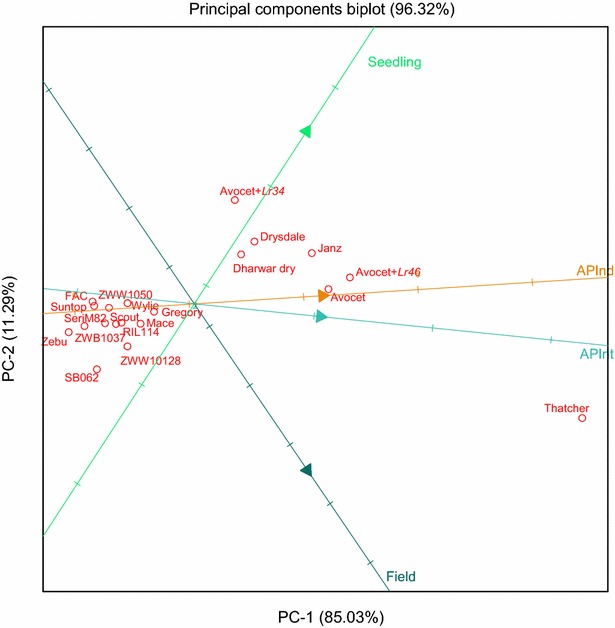
Biplot displaying results from principal component analysis using leaf rust response obtained in the following experiments: seedling (standard glasshouse), adult plant integrated (*APInt*) under accelerated growth conditions (flag-2 leaf), adult plant independent (*APInd*) under accelerated growth conditions (flag-2 leaf) and in the field (96 days after sowing). The displayed principle components (i.e. PC1 and PC2) account for 96.32 % of the variation

Results from PCA displayed in the biplot (Fig. 2) revealed a high correlation between both adult plant experiments conducted under AGC, where the adult plant integrated experiment appeared to be slightly more correlated to the field disease response. The field response was moderately correlated with the adult plant independent experiments performed under AGC (Fig. 2). Notably, only a weak correlation was observed between field and seedling response (Fig. 2).

## Discussion

This study presents a novel method that permits rapid phenotyping for APR to LR in wheat by exploiting AGC to speed up plant development and involves two sequential inoculations to detect APR. Characterization of a panel of 21 wheat genotypes revealed that the LR response displayed under AGC was indicative of levels expressed by adult plants grown in the field. Phenotyping for APR to LR can be completed within just 7 weeks and performed all-year-round, thus provides a useful tool to accelerate breeding and research aiming to develop rust resistant cultivars.

### Detection of APR to LR under AGC

Of the 21 spring wheat genotypes evaluated, 7 were determined to carry APR to LR, including; Avocet, Avocet + *Lr34*, Avocet + *Lr46*, Janz, Lang, Drysdale and Dharwar dry. These genotypes were considered susceptible in the seedling experiment, but displayed resistance in adult plant experiments. Genotypes known to carry APR genes, in particular *Lr13*, *Lr34* and *Lr46*, consistently displayed resistance at the adult pant stage under AGC—similar to levels displayed in the field. For instance, both Janz and Lang carry seedling gene *Lr24* and APR gene *Lr34* in combination; however *Lr24* is not effective against the pathotype used in this study. Therefore, these genotypes displayed a susceptible response in the seedling experiment, but a MRMS response under AGC at the adult plant stage, likely due to expression of *Lr34*. In some genotypes, the expression of *Lr34* was likely masked by the presence of effective seedling resistance genes, such as *Lr13* in EGA Gregory and *Lr17a* in EGA Wylie. Another good example of APR expression under AGC was observed for Avocet and the Avocet near-isogenic lines for *Lr34* (i.e. Avocet + *Lr34*) and *Lr46* (i.e. Avocet + *Lr46*). Notably, Avocet carries race specific APR gene *Lr13*, which is effective against the pathotype used in this study. The RMR response displayed by Avocet indicated that *Lr13* was successfully detected in the adult plant AGC experiments. In the Avocet background (*Lr13*), the addition of *Lr34* and *Lr46* enhanced the levels of resistance displayed in the adult plant experiments. This indicates the additive effect of APR genes can be detected under AGC. However, to detect the effectivity of the APR against different races the developed method can also be applied by conducting multiple screens using different pathotypes.

### Disease response under AGC is related to field-based measures

The GS of plants evaluated under AGC ranged between tillering to booting stage at time of inoculation with *P. triticina* and plants displayed adult plant phenotypes. This aligns well with previous studies on wheat that report early expression of APR to YR at mid-tillering growth stages in the field [30] and at the stem elongation stage in plants grown under controlled environment [15]. Regression analyses for the panel revealed that the flag-2 leaf expressed levels of APR most similar to those observed in the field. The upper-most infected leaf (i.e. flag leaf) displayed increased susceptibility to the pathogen in comparison to lower leaves. Thus, it appears APR is best expressed in ‘older’ leaves (that are more aged) compared to ‘younger’ leaves.

In the field, the inoculum pressure fluctuates due to infection cycles of rust urediniospores and weather conditions. One of the advantages of phenotyping under AGC is the application of inoculum can be controlled. It might be expected that the inoculum concentration applied under AGC using a single inoculation would correlate better with disease assessment performed early in the season (i.e. low disease pressure) as opposed to late in the season (i.e. high disease pressure). However, our results under AGC correlated well with measurements early in the season (i.e. 70 DAS) and late in the season (i.e. 96 DAS). It is feasible that phenotyping based on IT on a single leaf using a controlled single inoculation is indicative of factors important for reducing overall disease severity in the field under polycyclic conditions; such as pustule size and infection frequency.

### Importance of temperature and light to detect APR under AGC

AGC involves constant light and temperature regimes during the early plant growth phase to achieve adult plant stage rapidly. However, to assist a successful infection, diurnal light and temperature regime was implemented post-inoculation until disease assessment. Post-inoculation conditions are important for a successful host-pathogen interaction and become more important when plants are raised and inoculated in an artificial environment, such as the AGC adopted in this study. As discussed above, plant growth stage, along with temperature and light (i.e. quantity and quality) are considered key factors determining disease development [15].

All known *Lr* genes are sensitive to fluctuating post-inoculation temperatures, for instance expression of *Lr13* at the adult growth stage [16]. In the present study, plants were grown under a 12 h cycling temperature regime of 22/17 °C. This temperature enabled rapid plant growth, and importantly, provided healthy plants prior to inoculation. Notably, this falls within the optimal temperature range for LR development (i.e. 10–25 °C) [31]. Under AGC, a warmer growing temperature (e.g. >24 °C) can compromise plant health, which is critical if plants are to be subjected to disease assays. The increase or decrease in temperature can also influence latent period [16, 32]. The fluctuations in latent period are critical in wheat rust infections and AGC could serve as a tool to study the latent period under different temperature regimes.

Light is another key component of the rapid phenotyping method, where it not only affects plant photosynthetic activity, but also plays a role in disease development. Under AGC, wheat plants were grown under constant (24 h) light to quickly obtain adult plants. The importance of light influencing disease development both pre- and post-inoculation has been previously reported for both LR and YR in wheat [33]. We employed a diurnal (12 h) photoperiod post-inoculation until disease assessment. High quality light is important for disease development, particularly for good sporulation [34]. In addition, the diurnal light appears to be important, as constant (24 h) light can impede pathogen development, thus reducing the ability to differentiate between resistant and susceptible genotypes (unpublished data).

## Conclusion

Breeding for rust resistance requires a continuous effort to stay ahead of the rapidly evolving pathogen. This requires robust phenotypic screening and ongoing deployment of new resistance genes. The method reported in this study provides a great tool for detecting APR to LR at levels similar to those observed in the field. It can be scaled-up for screening large numbers of fixed lines and segregating populations, similar to that reported for YR in wheat [15]. Using this technique, it is possible to conduct up to seven consecutive screens annually, compared to just one in the field. It is possible to phenotype APR prior to anthesis under AGC, as genotypes inoculated at or beyond GS30 display resistance representative of adult plants. Assessing plants at early growth stages can enable selection of desirable gene combinations for APR and crossing of the selected plants in the same plant generation. This strategy would reduce time required for moving APR genes into adapted germplasm (from donor sources) or combining traits in top crosses in breeding programs.

## Additional file

10.1186/s13007-016-0117-7 Mean leaf rust response including infection type and host response for the panel of 21 spring wheat genotypes evaluated in the following experiments: seedling (standard glasshouse), adult plant integrated and adult plant independent under accelerated growth conditions (AGC), and in the field. The disease response for the seedling and adult plant AGC experiments was collected using the 0–4 Stakman scale whereas, the disease response in the field was collected using the modified Cobb scale, which was used to calculate coefficient of infection. A dash (-) indicates data is unavailable or unknown.

## References

[1] Hawkesford MJ, Araus J-L, Park R, Calderini D, Miralles D, Shen T, Zhang J, Parry MAJ (2013). Prospects of doubling global wheat yields. Food Energy Secur.

[2] Samborski D. Wheat leaf rust. In: Roelfs AP, Bushnell WR, editors. The cereal rusts-II. 1985. p. 58–76.

[3] Huerta-Espino J, Singh RP, Germán S, McCallum BD, Park RF, Chen WQ, Bhardwaj SC, Goyeau H (2011). Global status of wheat leaf rust caused by *Puccinia triticina*. Euphytica.

[4] Everts KL, Leath S, Finney PL (2001). Impact of powdery mildew and leaf rust on milling and baking quality of soft red winter wheat. Plant Dis.

[5] Bolton MD, Kolmer JA, Garvin DF (2008). Wheat leaf rust caused by *Puccinia triticina*. Mol Plant Pathol.

[6] Murray G, Brennan J (2009). Estimating disease losses to the Australian wheat industry. Australas Plant Pathol.

[7] Pink DC (2002). Strategies using genes for non-durable disease resistance. Euphytica.

[8] Park RF, Mohler V, Nazari K, Singh D (2014). Characterisation and mapping of gene *Lr73* conferring seedling resistance to *Puccinia triticina* in common wheat. Theor Appl Genet.

[9] Caldwell RM. Breeding for general and/or specific plant disease resistance. In: Finlay KW, Shepherd KW, editors. Proceedings of the Third International Wheat Genetics Symposium. Australian Academy of Sciences, Canberra: Australia; 1968. p. 263–72.

[10] Ellis JG, Lagudah ES, Spielmeyer W, Dodds PN (2014). The past, present and future of breeding rust resistant wheat. Front Plant Sci.

[11] McIntosh RA, Wellings CR, Park RF (1995). Wheat rusts: an atlas of resistance genes.

[12] Bansal UK, Hayden MJ, Venkata BP, Khanna R, Saini RG, Bariana HS (2008). Genetic mapping of adult plant leaf rust resistance genes *Lr48* and *Lr49* in common wheat. Theor Appl Genet.

[13] Lagudah ES, McFadden H, Singh RP, Huerta-Espino J, Bariana HS, Spielmeyer W (2006). Molecular genetic characterization of the *Lr34/Yr18* slow rusting resistance gene region in wheat. Theor Appl Genet.

[14] Moore JW, Herrera-Foessel S, Lan C, Schnippenkoetter W, Ayliffe M, Huerta-Espino J (2015). A recently evolved hexose transporter variant confers resistance to multiple pathogens in wheat. Nature genetics..

[15] Hickey LT, Wilkinson PM, Knight CR, Godwin ID, Kravchuk OY, Aitken EAB (2012). Rapid phenotyping for adult-plant resistance to stripe rust in wheat. Plant Breed.

[16] Kaul K, Shaner G (1989). Effect of temperature on adult-plant resistance to leaf rust in wheat. Phytopathology.

[17] Risk JM, Selter LL, Krattinger SG, Viccars LA, Richardson TM, Buesing G (2012). Functional variability of the *Lr34* durable resistance gene in transgenic wheat. Plant Biotechnol J.

[18] Singh D, Macaigne N, Park RF (2013). *Rph20*: adult plant resistance gene to barley leaf rust can be detected at early growth stages. Eur J Plant Pathol.

[19] Hickey LT, Dieters MJ, DeLacy IH, Kravchuk OY, Mares DJ, Banks PM (2009). Grain dormancy in fixed lines of white-grained wheat (*Triticum aestivum* L.) grown under controlled environmental conditions. Euphytica.

[20] Park RF, Bariana HS, Wellings CR, Wallwork H (2002). Detection and occurrence of a new pathotype of *Puccinia**triticina* with virulence for *Lr24* in Australia. Aust J Agric Res.

[21] Stakman E, Stewart D, Loegering W (1962). Identification of physiologic races of *Puccinia graminis var*. *tritici*.

[22] Zadoks JC, Chang TT, Konzak CF (1974). A decimal code for the growth stages of cereals. Weed Res.

[23] Peterson RF, Campbell A, Hannah A (1948). A diagrammatic scale for estimating rust intensity on leaves and stems of cereals. Can J Res.

[24] Loegering W (1959). Model of recording cereal rust data.

[25] Ziems LA, Hickey LT, Hunt CH, Mace ES, Platz GJ, Franckowiak JD (2014). Association mapping of resistance to *Puccinia hordei* in Australian barley breeding germplasm. Theor Appl Genet.

[26] Singh D, Park R, editors. Inheritance of adult plant resistance to leaf rust in four European winter wheat cultivars. In: 11th international wheat genetics symposium 2008 proceedings; 2008: Sydney University Press, Sydney

[27] Wellings C, Bariana H, Bansal U, Park R. Expected responses of Australian wheat and triticale varieties to the cereal rust diseases in 2012. Cereal Rust Report, Season. 2012;10(1):1–5.

[28] Pretorius ZA, Wilcoxson RD, Long DL, Schaffer JF (1984). Detecting wheat leaf rust resistance gene *Lr13* in seedlings. Plant Dis.

[29] Kloppers F, Pretorius Z. Expression and inheritance of leaf rust resistance gene *Lr37* in wheat seedlings. Cereal Res Commun. 1994;22(1–2):91–7.

[30] Park R, Rees R, editors. The epidemiology and control of stripe rust of wheat in the summer rainfall area of Eastern Australia. Second national stripe rust workshop, Wagga Wagga, New South Wales, Australia; 1987

[31] Dyck P, Johnson R (1983). Temperature sensitivity of genes for resistance in wheat to *Puccinia recondita*. Can J Plant Pathol.

[32] Eversmeyer M, Kramer C, Browder L (1980). Effect of temperature and host: parasite combination on the latent period of *Puccinia recondita* in seedling wheat plants. Phytopathology.

[33] De Vallavieille-Pope C, Huber L, Leconte M, Bethenod O (2002). Preinoculation effects of light quantity on infection efficiency of *Puccinia striiformis* and *P. triticina* on wheat seedlings. Phytopathology.

[34] Roelfs A, Huerta-Espino J, Marshall D. Barley stripe rust in Texas. Plant Dis. 1992;76:538.

[35] Hayes HK, Ausemus E, Stakman E, Bailey C, Wilson H, Bamberg R et al. Thatcher wheat. Minn Bull. 1936;325:1–39.

[36] Fitzsimmons R, Martin R, Wrigley C (1983). Australian wheat varieties: identification according to plant, head and grain characteristics.

[37] Lillemo M, Singh RP, Huerta-Espino J, Chen XM, He ZH, Brown JKM, Buck HT, Nisi JE, Salomón N (2007). Leaf rust resistance gene *Lr34* is involved in powdery mildew resistance of cimmyt bread wheat line Saar. Wheat production in stressed environments. Developments in plant breeding.

