# Supplementary material for: A rapid phenotyping method for adult plant resistance to leaf rust in wheat
Source: Plant Methods. 2016 Mar 2;12:17. doi: 10.1186/s13007-016-0117-7 (PMC4776422; doi:10.1186/s13007-016-0117-7)
Supplement: Supplementary file 1 — 10.1186/s13007-016-0117-7 Mean leaf rust response including infection type and host response for the panel of 21 spring wheat genotypes evaluated in the following experiments: seedling (standard glasshouse), adult plant integrated and adult plant independent under accelerated growth conditions (AGC), and in the field. The disease response for the seedling and adult plant AGC experiments was collected using the 0–4 Stakman scale whereas, the disease response in the field was collected using the modified Cobb scale, which was used to calculate coefficient of infection. A dash (-) indicates data is unavailable or unknown. [file 13007_2016_117_MOESM1_ESM.doc]

**Table S1** Mean leaf rust response including infection type and host response for the panel of 21 spring wheat genotypes evaluated in the following experiments: seedling (standard glasshouse), adult plant integrated and adult plant independent under accelerated growth conditions (AGC), and in the field. The disease response for the seedling and adult plant AGC experiments was collected using the 0-4 Stakman scale whereas, the disease response in the field was collected using the modified Cobb scale, which was used to calculate coefficient of infection. A dash (-) indicates data is unavailable or unknown.

| Genotypes | Seedling | | Mean leaf rust response adult plants | | | | | | |
| --- | --- | --- | --- | --- | --- | --- | --- | --- | --- |
|  |  |  | Integrated | | Independent | | Field | | |
|  | Infection type | Host response | Infection type | Host response | Infection type | Host response | Severity | Host response | Coefficient of infection |
| Thatcher | 4 | HS | 4 | S | 4 | S | 60 | S | 60 |
| Avocet | 4 | HS | 12 | RMR | 12 | RMR | 50 | MRMS | 30 |
| Avocet+*Lr34* | 3++ | S | 12- | RMR | ;n12- | RMR | 20 | MRR | 6 |
| Avocet+*Lr46* | 3+ | S | 2++ | MR | 12+ | MR | 40 | MRMS | 24 |
| Dharwar dry | 3+ | S | ;1 | R | ;n1 | R | 30 | MRMS | 18 |
| Drysdale | 3+ | S | 1- | R | 12 | RMR | 50 | MRR | 15 |
| Janz | 3+ | S | 23 | MRMS | 23 | MRMS | 30 | MRMS | 18 |
| Lang | 3+ | S | 23 | MRMS | 2+3 | MRMS | - | - | - |
| EGA Gregory | 12+ | R | ;n1 | R | ;n1- | R | 40 | MRR | 12 |
| EGA Wylie | ;12+ | R | ;1 | R | ;0 | HR | 30 | MRR | 9 |
| FAC10-16-1 | ;12+ | R | ;0 | HR | ;0 | HR | 30 | RMR | 9 |
| Mace | ;12+ | R | ;n1- | R | ;n1= | R | 40 | MRR | 12 |
| RIL114 | ;n1- | R | ;n1- | R | ;n | HR | 40 | MRR | 12 |
| SB062 | ;0 | R | ;0 | HR | ;0 | HR | 30 | MRMS | 18 |
| Scout | ;1+ | R | ;n1- | R | ;n1- | R | 40 | MRR | 12 |
| SeriM82 | ;n1- | R | ;0 | HR | ;n | HR | 40 | MRR | 12 |
| Suntop | ;n1= | R | ;n | HR | ;0 | HR | 30 | MRR | 9 |
| Zebu | ;0 | R | ;0 | HR | ;0 | HR | 30 | MRR | 9 |
| ZWB10-37 | ;0 | R | ;n | HR | ;0 | HR | 30 | MRR | 9 |
| ZWW10-50 | ;12+ | R | ;0 | HR | ;n1= | R | 30 | MR | 12 |
| ZWW10-128 | ;n1- | R | ;0 | HR | ;n | R | 30 | MRMS | 18 |
